# Acute Kidney Injury: From Diagnosis to Prevention and Treatment Strategies

**DOI:** 10.3390/jcm9061704

**Published:** 2020-06-02

**Authors:** Joana Gameiro, José Agapito Fonseca, Cristina Outerelo, José António Lopes

**Affiliations:** Department of Medicine, Division of Nephrology and Renal Transplantation, Centro Hospitalar Lisboa Norte, EPE, Av. Prof. Egas Moniz, 1649-035 Lisboa, Portugal; jose.nuno.agapito@gmail.com (J.A.F.); cristinaouterelo@gmail.com (C.O.); jalopes93@hotmail.com (J.A.L.)

**Keywords:** acute kidney injury, prevention, diagnosis, treatment

## Abstract

Acute kidney injury (AKI) is characterized by an acute decrease in renal function that can be multifactorial in its origin and is associated with complex pathophysiological mechanisms. In the short term, AKI is associated with an increased length of hospital stay, health care costs, and in-hospital mortality, and its impact extends into the long term, with AKI being associated with increased risks of cardiovascular events, progression to chronic kidney disease (CKD), and long-term mortality. Given the impact of the prognosis of AKI, it is important to recognize at-risk patients and improve preventive, diagnostic, and therapy strategies. The authors provide a comprehensive review on available diagnostic, preventive, and treatment strategies for AKI.

## 1. Introduction

Acute kidney injury (AKI) is a frequent diagnosis with an incidence that varies from 5.0% to 7.5% in hospitalized patients and that reaches up to 50–60% in critically ill patients [1,2,3,4,5,6]. AKI is characterized by an acute decrease in renal function that can be multifactorial in its origin and is associated with complex pathophysiological mechanisms [1,7].

In the short term, AKI is associated with an increased length of hospital stay, health care costs, and in-hospital mortality, and its impact extends into the long term, with AKI being associated with increased risks of cardiovascular events, progression to chronic kidney disease (CKD), and long-term mortality [8].

The incidence of AKI has increased in the past few decades, which might reflect the impact of the increased recognition of this diagnosis and improvements in patient care, namely through improvements in dialytic care, the availability of less nephrotoxic drugs, and a decrease in the use of dopamine and diuretics [1,9]. Mortality rates have declined in critically ill patients with AKI, but mortality rates are still significantly high and increase with AKI severity, specifically in dialysis-requiring AKI [6,9,10,11]. 

AKI survivors are at increased risk of developing CKD, which defined by the persistence of kidney disease for a period of more than 90 days [8]. Additionally, investigators now consider that AKI and CKD are part of a disease continuum instead of separate entities [8]. Indeed, the term acute kidney disease (AKD) has been recently proposed to define the continuing pathological processes and adverse events developing after AKI [12].

AKD is defined as an acute or subacute damage and/or a loss of kidney function for a duration of between 7 and 90 days after exposure to an AKI-initiating event [12]. This highlights the importance of renal recovery, as recovery within 48 h is typically associated with the rapid reversal of AKI, as well as the impact of AKD on pre-existing CKD in increasing the risk for kidney disease progression [12].

Given the impact of the prognosis of AKI, it is important to enhance the recognition of at-risk patients and to improve preventive, diagnostic, and therapeutic strategies. The authors provide a comprehensive literature review on available preventive and therapeutic strategies for AKI.

## 2. Diagnosis of AKI

The currently widespread AKI classification was developed by the Kidney Disease Improving Global Outcomes (KDIGO) work group in 2012 and defines AKI as an increase in the serum creatinine (SCr) level to at least 0.3 mg/dL within 48 h, an increase in SCr to more than 1.5 times the baseline (which is known or presumed to have occurred within the prior 7 days), or a urine output (UO) decrease to less than 0.5 mL/kg/h for 6 h [13]. This classification also stratifies different stages of AKI severity and provides criteria that could be applied in clinical activity and investigation [14] (Table 1).

The current definition relies on SCr and UO, which are imperfect markers with significant limitations, namely that these do not account for the duration or cause of AKI [15]. SCr is an insensitive marker because it is altered by factors affecting its production (age, gender, diet, muscle mass, and sepsis), dilution (fluid administration), elimination (previous renal dysfunction), and secretion (medications). Thus, SCr cannot be used as an accurate estimate of glomerular filtration rate (GFR) in the non-steady state, and it underestimates the degree of dysfunction due to reduced muscle mass, increased catabolism, or positive fluid balance in critical patients. Additionally, it often takes two-to-three days before SCr is elevated after a renal insult when renal injury occurs in the setting of appropriate renal reserve, meaning that other nephrons increase function to compensate for injured nephrons, and so SCr may not change despite actual structural damage [16,17].

Though UO is an early marker for AKI, it also relies on patient’s volemic and hemodynamic status and the use of diuretics, and so it is difficult to assess without a urinary catheter and its usefulness relies on an hourly assessment that is time consuming [15,17,18].

Recently, potential urinary and serum biomarkers of AKI have been identified, namely cystatin-C, neutrophil gelatinase associated lipocalin (NGAL), N-acetyl-glucosaminidase (NAG), kidney injury molecule 1 (KIM-1), interleukin-6 (IL-6), interleukin-8 (IL-8), interleukin 18 (IL-18), liver-type fatty acid-binding protein (L-FABP), calprotectin, urine angiotensinogen (AGT), urine microRNAs, insulin-like growth factor-binding protein 7 (IGFBP7), and tissue inhibitor of metalloproteinases-2 (TIMP-2) [19,20,21,22,23,24,25,26,27,28,29]. Both NGAL and IGFBP7 with TIMP-2 are the most promising markers that have been validated in multiple settings. However, their increased cost and a lack of substantial evidence of improvement of outcomes are important limitations for their widespread clinical use [30,31].

These markers reflect different stages of the pathophysiology of AKI, so, the use of a panel of several biomarkers covering different phases of this syndrome might provide a better early diagnostic tool for AKI, as well as providing targets for future treatments [27,28,30,32,33,34,35]. 

## 3. Risk factors for AKI

Both patient susceptibilities and exposures are risk factors for AKI [11,36,37] (Table 2).

Patient age is an important non-modifiable risk factor, as the loss of renal reserve and the physiologic decline of GFR may place older patients at risk for AKI [38,39,40,41,42]. 

CKD patients possess a loss of autoregulation, abnormal vasodilation, susceptibility to antihypertensive agents, and nephrotoxins, and the side effects of medication contribute to the development of AKI [42]. Moreover, AKI and CKD have been described as interconnected syndromes because AKI leads to the worsening of CKD and CKD predisposes one to AKI [42,43]. CKD also limits renal recovery after AKI [44].

Patient comorbidities such as diabetes mellitus, hypertension, cardiovascular disease, chronic liver disease, and chronic obstructive pulmonary disease have also been identified as important AKI predictors [11,13,36,37,40,45,46]. Given the increasing incidence of HIV-infected patients in past few decades, HIV infection is also a risk factor that predisposes patients to AKI [47,48].

Exposure to sepsis, surgery, nephrotoxins, and shock are specific modifiable factors that contribute to AKI [1,13]. Indeed, large cohort studies focusing on critically ill patients have reported that the two most important causes of AKI are sepsis and surgery [6,49]. 

Additionally, recent research has reported that other factors like hyperuricemia, hypoalbuminemia, obesity, anemia, and hyperglycemia have been associated with an increased risk of AKI [49,50,51,52,53,54,55,56,57,58,59,60,61,62,63,64,65,66,67,68,69].

## 4. Causes and Assessment of AKI

AKI is a complex syndrome involving numerous pathophysiological processes that include pre-renal AKI, acute tubular necrosis, acute interstitial nephritis, acute glomerular diseases, and acute obstructive nephropathy [54].

These causes can be systematized into three groups, namely prerenal AKI, which accounts for up to 60% of cases and results from the functional adaptation to hypoperfusion of structurally normal kidneys; intrinsic renal AKI, which results from structural damage to any component of the renal parenchyma and accounts for up to 40% of cases; and, less frequently, postrenal AKI, which results from urinary tract obstruction [55,56]. The causes are summarized in Table 3. 

Essentially, the majority of causes of AKI are actually not renal-specific because the kidneys are highly sensitive to any systemic upset [55]. Indeed, the most common causes being septic shock, post major surgery, cardiogenic shock, and hypovolemia highlight this fact [57].

Most cases are multifactorial, and, following the inciting event causing kidney injury, numerous pathophysiologic pathways occur, including hemodynamic instability, microcirculatory dysfunction, tubular cell injury, tubular obstruction, renal congestion, microvascular thrombi, endothelial dysfunction, and inflammation [55,56,58,59,60].

The assessment of the cause of AKI must include a careful history, including medications and exposures, as well as a thorough physical examination. The assessment of fluid status and the presence of signs and symptoms of acute or chronic heart failure, infection, and urinary tract obstruction must be included in a first approach [54,61].

Laboratory evaluation should comprise SCr, urea, electrolytes, complete blood count, liver function tests, glucose level, bone profile, urine analysis, and microscopic examination, and a renal ultrasound must be performed to exclude obstruction. Urine output should be measured. A chest x-ray can provide evidence of a potential cause, such as pneumonia or vasculitis, but it can also prove useful in volume overload evaluation [54,61].

Less frequent causes of AKI such as vasculitis, glomerulopathy, and hemolytic uremic syndrome should be considered in the presence of fever, rash, joint pains, pulmonary infiltrates, abnormal urine analysis, thrombocytopenia, and hemolytic anemia when significant dehydration, hypotension, nephrotoxins, and obstruction have been excluded [54,61]. Thus, the determination of the cause of AKI must be completed with an assessment of antineutrophil cytoplasmic antibodies (ANCA), anti-glomerular basement membrane antibodies (anti-GBM), antinuclear antibodies (ANA), anti-double stranded DNA (anti-dsDNA) antibodies, complement factors, rheumatoid factor, antistreptolysin O titer (ASOT), cryoglobulins, serum electrophoresis, immunoglobulins, serum free light chains, hepatitis, and HIV serology [54,61] (Table 4).

## 5. Treatment of AKI

The therapeutic strategies for AKI based on the KDIGO guidelines and bundles of care are limited and mostly supportive [13].

The clinical approach should begin by hemodynamic stabilization, the early identification of complications of AKI, the identification of its cause, and its treatment [62]. Hemodynamic stabilization is of critical significance because autoregulation mechanisms are impaired in AKI [63].

Particular attention should be given to medications with renal toxicity, which should be discontinued, and dose adjustment according to renal function to avoid underdosing or adverse effects [62,64]. Additionally, in septic patients, prompt initiation of antibiotics is crucial [65].

It is important to rapidly identify and treat other complications in the therapeutic approach for the patient with AKI, such as hyperkalemia, metabolic acidosis, anemia, and fluid overload [13]. 

During the course of AKI, it is also recommended to prevent infection and start stress-ulcer prophylaxis [13].

### 5.1. Fluid Therapy

Fluid balance should be individualized, although the optimal fluid to this effect is undetermined. The titration of fluids is complex and requires the careful monitoring of patient’s volemia [63]. Hypovolemia reduces renal blood flow, but AKI patients are also at risk for volume overload [55]. Furthermore, a positive fluid balance is independently associated with increased mortality in AKI patients and contributes to worse outcomes in critically ill patients [66,67,68]. Goal-directed therapy guided by the assessment of fluid responsiveness appears to be associated with better outcomes [69,70]. 

Different types of fluids have different mechanisms of action. While colloids, such as albumin or starches, rely on oncotic gradients and selectively expand the extracellular space, crystalloids—namely saline, Ringer’s lactate or PlasmaLyte—equilibrate across intravascular and extravascular spaces [63,71].

Albumin appears to be relatively safe, but a consistent survival advantage compared with crystalloids has not been demonstrated [72,73,74]. The Saline Versus Albumin Fluid Evaluation (SAFE) trial reported that there was no renal or mortality benefit in patients who received albumin, but less total volume was required for resuscitation, which could be attractive in reducing positive fluid balance [72]. Furthermore, in the Colloids Versus Crystalloids for the Resuscitation of the Critically Ill (CRISTAL) trial, there was a decrease in need for mechanical ventilation, the need for vasopressors, and the mortality at 90-days in patients who received colloids [75].

Thus, albumin may prove important when large volumes of fluids are anticipated. Indeed, the recent Surviving Sepsis Campaign recommendations state that when patients require substantial amounts of crystalloids, albumin might be additionally used [76]. Still, there are no data supporting the routine use of colloids for volume resuscitation [77]. 

The administration of albumin in combination with terlipressin is also beneficial in specific conditions, such as cirrhotic patients and cardiac surgery patients with hypoalbuminemia in which the preoperative administration of albumin reduces the risk of postoperative AKI [78,79]. Nevertheless, caution must be taken in patients with traumatic brain injury, in which albumin has been associated with an increased mortality risk and should be avoided [72]. Some concerns have been raised on the use of hyperoncotic albumin in septic shock patients who are volume depleted and have increased vascular permeability, as it might increase AKI risk by promoting intracellular dehydration to expand volume [80,81].

Large randomized trials have reported higher incidences of AKI in patients treated with starches, which appears to be due to osmotic nephrosis with proximal tubule vacuolization and swelling. [63,82,83] The use of starches should thus be avoided [82].

There are less data concerning the use of gelatins and AKI, though observational data suggest these might contribute to AKI due to osmotic nephrosis [84]. Therefore, the use of gelatins is also not recommended in AKI [13,63].

Saline is the most frequently used crystalloid in critically ill patients [63,85]. Nevertheless, the administration of large volumes can cause hyperchloremia and metabolic acidosis. Hyperchloremia can lead to renal vasoconstriction and a consequent reduction in glomerular filtration [71,86]. 

Studies comparing different types of crystalloids in critically ill patients at risk of AKI have demonstrated conflicting results [85,86,87,88,89]. Yunos reported that a chloride-restrictive strategy was associated with a significant decrease in the incidence of AKI in critically ill patients [88]. The Saline versus PlasmaLyte for intensive care unit (ICU) Fluid Therapy (SPLIT) trial did not find differences in the rates of AKI or mortality in a population of mostly postoperative patients who received modest volumes for resuscitation [90,91]. In the Isotonic Solutions and Major Adverse Renal Events Trial (SMART) and in the Saline against Lactated Ringer’s or PlasmaLyte in the Emergency Department (SALT-ED), trial there were reductions in adverse kidney outcomes within 30 days in patients who received buffered solutions [85,92]. Though it is theorized that AKI occurs due to hyperchloremia only when large volumes are administered, a recent study of critically ill patients receiving large volumes of fluid did not demonstrate an association between chloride load and AKI risk after adjusting for disease severity [93]. Thus, saline remains the preferred solution for volume resuscitation, though chloride concentrations should be monitored [13,55,62].

### 5.2. Vasopressor Drugs

After volume resuscitation, vasopressor support should be considered to maintain renal perfusion in order to avoid positive fluid balance. In patients with AKI, the median blood pressure target should be higher than 65 mmHg to ensure accurate renal perfusion [13,94,95,96].

In vasodilatory states, noradrenaline is the recommended first-line vasopressor [76,97]. Noradrenaline improves microcirculatory flow by increasing perfusion pressure above the autoregulation threshold in hypotensive patients, but in high doses, it can cause a decrease in the flow by excessive vasoconstriction [98]. Thus, the current recommendations suggest to administer the lowest dose to achieve the blood pressure target and keep adequate perfusion parameters [98].

Vasopressin and terlipressin are effective alternatives for raising blood pressure, though their benefit on kidney function or mortality comparing to noradrenaline has not been demonstrated [99,100,101,102]. Angiotensin II has shown promising results on patient outcomes in recent studies, namely by improving survival and renal function recovery [103]. Nevertheless, further studies are still required to recommend the routine use of angiotensin II.

In contrary to previous beliefs, there is no evidence for a “renal dose” dopamine in AKI management, because there is no association between dopamine use in AKI and improvement in survival or renal function [104,105,106,107]. Fenoldopam has a similar hemodynamic renal effect to that oe low-dose dopamine and had been demonstrated to decrease systemic vascular resistance whilst increasing renal blood flow to the kidney [108,109]. Despite promising studies, there has been no conclusive evidence of the beneficial effect of fenoldopam in the management of AKI, and further investigation is warranted [110,111].

### 5.3. Diuretics

The use of diuretics is only recommended to manage fluid overload and electrolyte disturbances in AKI [13,112]. Based on pathophysiology studies, it was previously thought that loop diuretics might protect the loop of Henle from ischemia by decreasing its workload [112]. This has never been confirmed, and, on the contrary, it has been demonstrated that furosemide is not associated with clinical benefits in preventing AKI, decreasing the need for renal replacement therapy (RRT), renal recovery, or decreasing in-hospital mortality [113,114,115]. It is important to note that certain studies have associated the use of loop diuretics with an increased risk of mortality, which might be related to the delay in appropriate RRT start [112,115,116]. Additionally, a loop diuretic used in high doses may cause ototoxicity [112]. Therefore, the KDIGO guidelines do not recommend the use of diuretics to prevent AKI [13].

### 5.4. Drug Nephrotoxicity

Drug nephrotoxicity has been associated with 20–40% of AKI causes and can reach up to 60% in elderly patients [117,118]. Patients with underlying AKI or CKD, sepsis, acute or chronic liver failure, acute or chronic heart failure, pulmonary hypertension, malignancies, and exposure surgery are at increased risk for drug-induced nephrotoxicity [117].

The mechanisms of drug-induced nephrotoxicity are diverse and vary with different drug classes. (Table 3). Drugs can induce not only direct toxicity due to tubular injury, interstitial nephritis, glomerular injury, or obstructive nephropathy but also indirect nephrotoxicity associated with a decrease in renal blood flow [119]. Furthermore, drug-induced nephrotoxicity results from the combination of innate drug toxicity, altered renal hemodynamics, previous renal disease, and altered drug pharmacokinetics in critical illness [119,120].

Due to the associated reduced glomerular pressure, patients exposed to nonsteroidal anti-inflammatory drugs (NSAIDs), renin–angiotensin–aldosterone system blockers, high-dose systemic vasoconstrictors, or calcineurin inhibitors are at high-risk to develop prerenal AKI in the setting of altered systemic and renal hemodynamics or fluid loss; these also increase the risk for acute tubular injury [117].

Acute tubular necrosis (ATN) is dose-dependent and is the most common form of drug-induced AKI in the hospital setting [121]. The most common drugs associated with ATN are aminoglycosides, vancomycin, radiocontrast media, cisplatin, amphotericin B, foscarnet, and osmotically active agents [117,121].

Acute interstitial nephritis (AIN) causes up to 10% of AKI cases. AIN is an idiosyncratic reaction that is not dose-dependent [117,120]. Antimicrobials such as b-lactams, sulfa-based drugs and quinolones, anti-ulcer agents, anti-convulsants, and diuretics are the most common drugs associated with AIN [117,118,122].

Post-renal AKI due to crystal-induced luminal obstruction can occur in patients exposed to acyclovir, sulfa-based medications, ciprofloxacin, and methotrexate [121]. Less frequently, drug-induced glomerular disease can result from the administration of hydralazine, propylthiouracil, allopurinol, and penicillamine, all of which have been associated with development of ANCA vasculitis, mitomycin C, oral contraceptive agents, calcineurin inhibitors, antineoplastic agents, ticlopidine, and quinine, all of which can cause thrombotic microangiopathy [121].

Therefore, the prescription of drugs must be carefully considered to minimize toxicity. The KDIGO guidelines recommend the early discontinuation of potential nephrotoxic drugs, the avoidance of radiocontrast and other nephrotoxic drugs, and drug dose monitoring [13].

### 5.5. Other Therapeutic Strategies

Remote ischemic preconditioning is a technique that induces multiple short cycles of ischemia and reperfusion by cuff inflation [123]. This has been tested as a possible method to protect the kidneys from ischemia reperfusion injury, although there is conflicting evidence regarding the results in reducing AKI or mortality, and it is not recommended in clinical practice [124,125,126,127,128].

Levosimendan has both vasodilatory and inotropic actions [129]. It might improve kidney function by improving cardiac function but also through afferent arteriolar vasodilatation, though this benefit remains debatable [130]. In a recent meta-analysis, the use of levosimendan was associated with a decrease in AKI incidence and mortality in cardiac surgery patients, proving its potential use [131]. 

Several new potential therapeutic targets for AKI are currently being investigated and entering clinical trials. These include pathways involved in inflammation, fibrosis, mitochondrial function, oxidative stress, and hemodynamics. Though promising, further clinical trials are still required.

Reltecimod is a peptide antagonist of CD28 (co-stimulatory receptor) that acts as an immunomodulator and has been demonstrated to attenuate the systemic inflammatory response and decrease organ dysfunction in necrotizing soft tissue infections [132,133]. 

Due to the fact that active vitamin D has anti-proliferative and pro-differentiation actions, it might be that lower levels of vitamin D contribute to AKI [134,135]. Indeed, critically ill patients with vitamin D deficiencies have been reported to have higher rates of AKI and further progression to CKD [135,136,137]. Calcifediol and calcitriol are still under investigation as possible treatments in early AKI [138,139,140]. 

Alkaline phosphatase is an enzyme expressed along the proximal tubule and has the ability to reduce renal inflammation. The use of human recombinant alkaline phosphatase has been investigated in clinical trials as a potential anti-inflammatory drug that could attenuate kidney injury or promote renal regeneration [141,142,143]. A recent trial reported that, despite not affecting short-term kidney function, there was a long-term benefit in kidney function [144].

Teprasiran is under investigation as an apoptosis inhibitor and has had promising results in patients undergoing cardiac surgery at risk for AKI by demonstrating a reduction in major adverse kidney events at 90 days [145,146]. 

Intensive investigation in this area reflects the fact that, to date, there are no established pharmacotherapies for AKI. The most important measures to be applied in clinical practice remain the hemodynamic monitoring and administration of fluids and vasopressors, the eviction and avoidance of nephrotoxins, and the treatment of AKI complications (Table 4).

### 5.6. Renal Replacement Therapy

Conventional criteria for initiation of RRT in AKI are anuria, severe/refractory hyperkalemia, severe/refractory metabolic acidosis, refractory volume overload, severe azotemia, or clinical complications of uremia such as encephalopathy, pericarditis, or neuropathy [147,148]. 

There are different modalities of RRT that can be provided in cases of severe AKI, namely intermittent hemodialysis (HD), continuous RRT (CRRT), slow low-efficiency dialysis (SLED), or peritoneal dialysis (PD) [149]. Continuous RRT (CRRT) is the most common form of renal support provided to critically ill patients because it provides better volume control and acid–base and electrolyte correction while maintaining hemodynamic stability [147,148]. Though there is no sustained evidence reporting a difference in mortality between the use DH, SLED, or CRRT in AKI patients, there is a tendency for earlier renal recovery and decreased progression to CKD with CRRT use [150,151]. The KDIGO guidelines recommend the use of CRRT and HD as complementary therapies, although CRRT should be favored in hemodynamically unstable patients and in patients with increased intracranial pressure [13]. Ultimately, the choice between RRT modalities relies on patient clinical status, resource availability, and local expertise [149].

RRT is essential to maintain volume, electrolyte, acid-base, and uremic solute homeostasis in AKI patients. It is also theorized that RRT can modulate inflammation, which might prove crucial in septic patients, though this remains uncertain [147,152]. Nonetheless, RRT requires central venous dialysis catheter insertion, the exposure of blood to an extracorporeal circuit, and anticoagulation, and it can be associated with hemodynamic instability, which may contribute to delayed kidney recovery [147,152].

The timing to start RRT remains controversial. According to the KDIGO guidelines, RRT should be started when life-threatening changes in fluid, electrolyte, and acid-base balances exist, and it is recommended to consider the broader clinical context and trends of laboratory values when making the decision to start RRT [13]. It is also non-consensual if RRT can change patient outcomes or is merely a surrogate for the critical illness on patient outcomes [153,154,155,156]. 

Recent randomized clinical trials have evaluated the optimal timing to start RRT in critically ill AKI patients. The heterogeneity in definitions of timing and criteria has contributed to lack of a strong recommendation [153,154].

The Early Versus Late Initiation of RRT (ELAIN) was a single-center trial of 231 critically ill, mostly surgical AKI patients. In this study, Zarbock et al. defined the early group as starting RRT within 8 h of fulfilling KDIGO stage 2 AKI and elevated plasma NGAL levels, and they defined the delayed group as starting RRT within 12 h of developing KDIGO stage 3 AKI or in the presence of an absolute indication [157]. The early RRT group was associated with 15% less mortality, greater RRT independence, and less hospitalization days than the delayed RRT group. However, the delayed group included 9% of patients who did not start RRT due to the recovery of kidney function [157].

In contrast, in the Artificial Kidney Initiation in Kidney Injury (AKIKI) multicenter trial of 620 critically ill AKI patients, an early RRT start did not decrease mortality, with no differences noted in hospital stays and renal recovery, and the delayed group had greater RRT-free days and fewer incidences of catheter-related infections [158]. In this study, early RRT was defined as starting RRT within 6 h of fulfilling KDIGO stage 3 AKI, and delayed RRT was defined as RRT start only in response to the development of absolute indications. Additionally, only 51% in the delayed group started RRT [158]. 

Considering the contrasting results of these studies, another multicenter trial was conducted in 488 patients with septic shock and severe AKI; this was entitled Initiation of Dialysis Early versus Late in the Intensive Care Unit (IDEAL-ICU) [159]. In this study, Barbar et al. defined the early group as RRT start within 12 h of achieving the failure stage of the RIFLE (Risk, Injury, Failure, Loss of kidney function, and End-stage kidney disease) classification without life-threatening AKI complications, and they defined the delayed group as starting RRT after a delay of 48 h of achieving the failure stage of the RIFLE classification. The results of the IDEAL-ICU trial were consistent with AKIKI and also demonstrated no significant difference in mortality between groups [159].

Only the ELAIN trial provided evidence that suggested the benefit of an early RRT start, which has not been verified in most recent studies. Several factors might have contributed to this conflicting evidence. 

Firstly, the population studied, the AKI diagnostic criteria, and the definition of timing in each study were different. The ELAIN trial included a mixed ICU population but was mostly surgical patients, whereas the AKIKI and IDEAL trials included medical ICU patients with a higher proportion of sepsis. The ELAIN trial also included a significantly higher proportion of CKD patients (with a glomerular filtration rate of higher than 30 mL/min/1.73 m^2^) than the AKIKI and IDEAL trials (41% vs. 10% and 15%, respectively). Patients enrolled in the AKIKI and IDEAL trials were at least stage 3 KDIGO or failure on the RIFLE classification, while this was only the case for patients in the delayed group in the ELAIN trial. Thus, the inclusion of less severe patients in the ELAIN trial might have beneficially influenced the outcome. Interestingly, patients in the ELAIN trial received delayed RRT at a significantly earlier point than the delayed group in the AKIKI and IDEAL trials (25.5 vs. 57 and 51.5 h, respectively). 

Secondly, RRT modalities were different in these trials. All patients in the ELAIN trial were started on continuous RRT, and a combination of continuous and intermittent RRT techniques were prescribed in the AKIKI and IDEAL trials. Therefore, the disparities between RRT modalities might have influenced the hemodynamic assessment, treatment, and outcome of these patients.

Finally, a lower proportion of patients in the delayed group of the AKIKI and IDEAL trials initiated RRT than the delayed group of the ELAIN trial (51% and 62% vs. 91%, respectively). This suggests that a conservative approach to RRT start in response to persistent AKI or complications might be acceptable, as several patients with KDIGO stage 3 recovered renal function and ultimately did not require RRT.

The results of the ongoing Standard Versus Accelerated Initiation of Dialysis in Acute Kidney Injury (STARRT-AKI) and AKIKI 2 multicenter trials are highly anticipated in order to provide a more definitive answer to the problem of optimal timing for RRT start [160,161].

The timing of initiation has only been assessed in critically ill and the post-surgical settings, [162,163] and the inconsistent results have led to a perpetuation of the traditional recommendations of RRT start.

## 6. Prevention of AKI

In the absence of effective therapeutic interventions on established AKI and due to its significant on morbidity and mortality, we can only rely on AKI prevention and early diagnosis to reduce its incidence and detrimental consequences. 

On the other hand, it could be argued that risk assessment is futile because it is unclear which interventions for high-risk patients should be implemented and whether these interventions are actually effective. 

However, recent studies have suggested that the risk stratification of patients for AKI can allow for the employment of effective intervention and reduce the incidence of AKI, although there has been no evidence of benefit for long-term renal outcomes [164,165,166]. 

The recent Acute Disease Quality Initiative (ADQI) conference on “Quality Improvement for AKI” proposed that the range of care in AKI should be a continuum from risk assessment and prevention in the community setting, to AKI prevention in the hospital, to optimizing AKI management, and finally to the surveillance of AKD and the prevention of recurrent AKI and progression to CKD [167].

At least 50% of AKI episodes are believed to begin in the community setting, so health care professionals should identify at-risk patients (Table 2) and implement preventive interventions to decrease the incidence of AKI [168,169,170].

High-risk patients for AKI should have a kidney health assessment (KHA) every 12 months, at least 30 days before exposure and two-to-three days after exposure that carries AKI risk in order to define and modify their risk profile [167,171]. The KHA must include AKI and CKD history, blood pressure assessment, SCr level, urine dipstick, and medication list [167]. 

After an acute exposure to an AKI inciting event, namely nephrotoxic medications, radiocontrast, surgery, or acute disease, medications should be adjusted, further nephrotoxic exposures should be minimized, and AKI occurrence and its complications should be monitored [167].

At hospital admission, patients should also be screened for AKI risk [13,169,172]. In high-risk patients, the early correction of modifiable risk factors should be considered in order to prevent AKI occurrence [64,167,173].

The gold-standard of AKI prevention remains the optimization of hemodynamic and volume status, medication review such as the cessation of angiotensin-converting enzyme inhibitor/angiotensin receptor blocker (ACEi/ARB) and metformin, and the minimization of nephrotoxic exposure [13,174].

## 7. Follow-Up after AKI

For patients with AKI, the main goal should be the recovery to baseline kidney function in the shortest period of time in order to reduce duration and disease severity, thus highlighting the role of early diagnosis and prompt management [62,167,175].

To date, there remains no standardized AKI or AKD follow-up care, but several studies have reported low rates of nephrology follow-up after AKI across different settings. [176,177]. In the United States Renal Data System annual report of 2015, only 19% of patients had a nephrology follow-up at 12 months after an AKI hospitalization [178]. In another study, only 4% of patients were referred to a nephrologist at three months and only 9% at one year, though the mortality rate during this period was 22% [179].

The benefit of nephrology referral is uncertain, however, recent studies have suggested that in high-risk patients, an early nephrology referral may improve survival. Indeed, Harel et al. reported that only 41% of AKI patients had a nephrology follow-up and that this was associated with a 24% mortality reduction in two years of follow-up [180].

Determining which patients are at higher risk for CKD development after AKI is crucial. Risk factors for CKD after AKI include the severity, duration, and recurrence of AKI; the timing of renal recovery; advanced age; lower baseline renal function; diabetes mellitus; hypertension; chronic heart failure; hypoalbuminemia; proteinuria; chronic liver disease; and a higher Charlson comorbidity index [181].

The KDIGO guidelines and the ADQI consensus recommend that after an episode of AKI, patients should be followed by a nephrologist at least three months after the episode in order to assess kidney recovery and/or progression to CKD or progressive CKD [13,167]. The follow-up evaluation should include kidney function and proteinuria to assess prognosis and outcome, medication reconciliation, patient education to nephrotoxic avoidance, and the employment of strategies to prevent CKD progression [167].

Further research is warranted to identify high-risk patients, define timing for nephrology follow-up, and to develop strategies to improve patient outcomes.

## 8. Conclusions

Currently, there are no established pharmacotherapies for AKI. Treatment strategies for AKI comprise hemodynamic stabilization, the eviction of nephrotoxins, and the treatment of AKI complications. The gold-standard of AKI prevention includes identifying at-risk patients, optimizing hemodynamic and volume statuses, reviewing medication, and minimizing nephrotoxic exposure. Considering the significant prognostic impact of AKI, it is crucial to focus further research on AKI prevention and therapy.

## Figures and Tables

**Table 1:** Kidney Disease Improving Global Outcomes (KDIGO) classification.

Stage	SCr	UO
1	Increase in SCr ≥ 0.3 mg/dL (≥26.5 μmol/L) or increase in SCr ≥ 150% to 200% (1.5 to 1.9X)	<0.5 mL/kg/h (>6 h)
2	Increase in SCr > 200% to 300% (>2 to 2.9X)	<0.5 mL/kg/h (>12 h)
3	Increase in SCr > 300% (≥3X) or Increase in SCr to ≥4 mg/dL (≥353.6 μmol/L) or initiation of renal replacement therapy	<0.3 mL/kg/h (24 h) or anuria (12 h)

SCr: serum creatinine; UO: urine output.

**Table 2:** Risk factors for acute kidney injury (AKI).

AKI Risk Factors	
Older age	Shock
Diabetes	Sepsis
Hypertension	Nephrotoxins
Chronic kidney disease	(NSAIDs, ARB, ACEi, contrast)
Cardiovascular disease	Surgery
Chronic liver disease	Hyperuricemia
Chronic obstructive pulmonary disease	Hypoalbuminemia
HIV infection	Hyperglycemia
Obesity	Anemia

ACEi: angiotensin-converting enzyme inhibitor; ARB: angiotensin receptor blocker; NSAID: nonsteroidal anti-inflammatory drugs.

**Table 3:** Causes of AKI.

Causes of AKI		
**Pre-renal**	**Intrinsic**	**Post-renal**
*- Hypovolemia* Hemorrhage Volume depletion Renal fluid loss (over-diuresis) Third space (burns, peritonitis, muscle trauma	*- Tubular* Renal ischemia (shock, surgery, hemorrhage, trauma, bacteremia, pancreatitis, pregnancy) Nephrotoxic drugs (antibiotics, antineoplastic drugs, contrast media, organic solvents, anesthetic drugs, heavy metals) Endogenous toxins (myoglobin, hemoglobin, uric acid)	*- Extrarenal obstruction* Prostate hypertrophy Improperly placed catheter Bladder, prostate or cervical cancer Retroperitoneal fibrosis
*- Impaired cardiac function* Congestive heart failure Acute myocardial infarction Massive pulmonary embolism	*- Glomerular* Post-infectious glomerulonephritis Lupus nephritis IgA glomerulonephritis Infective endocarditis Goodpasture syndrome Wegener disease	
*- Systemic vasodilatation* Anti-hypertensive medications Gram negative bacteremia Cirrhosis Anaphylaxis	*- Interstitial* Infections (bacterial, viral) Medications(antibiotics, diuretics, NSAIDs, anti-ulcer agents)	*- Intrarenal obstruction* Nephrolithiasis Blood clots Papillary necrosis Drugs (acyclovir, methotrexate)
*- Increased vascular resistance* Anesthesia Surgery Hepatorenal syndrome NSAID medications Drugs that cause renal vasoconstriction (cyclosporine, ARB, ACEi)	*- Vascular* Large vessels (bilateral renal artery stenosis, bilateral renal vein thrombosis) Small vessels (vasculitis, malignant hypertension, atherosclerotic or thrombotic emboli, hemolytic uremic syndrome, thrombotic thrombocytopenic purpura)	

IgA: Immunoglobulin A.

**Table 4:** Preventive and treatment strategies for AKI.

**Prevention of Acute Kidney Injury**	
**Identify high-risk patients**	
Kidney health assessment in high risk patients	CKD history
	blood pressure assessment
	SCr level
	urine dipstick
	medication list
	Every 12 months
	30 days before exposure to AKI risk
	2–3 days after exposure to AKI risk
Discontinue and/or avoid nephrotoxins Optimize hemodynamic and volume status	
**Treatment of Acute Kidney Injury**	
Correction of Hypovolemia	Individualized fluid therapy
	Avoid positive fluid balance
	Isotonic Saline
	Albumin
Vasopressor support (MAP > 65 mmHg)	Noradrenaline
	Vasopressin
	Terlipressin
Discontinue nephrotoxins and Adjust drugs to renal function	NSAIDs
	ARBs, ACEis
	Contrast
	Metformin
	Aminoglycosides
	Vancomycin
Absolute indications for RRT	Severe/refractory hyperkalemia
	Severe/refractory, metabolic acidosis
	Refractory volume overload
	Clinical complications of uremia (encephalopathy, pericarditis or neuropathy)
**Investigate and Treat Acute Kidney Injury Cause**	
SCr, Urea, Electrolytes	ANCA antibodies, anti-GBM antibodies, ANA antibodies, anti-dsDNA antibodies
Complete blood count, Liver function tests, Glucose level, Bone profile	complement factors, rheumatoid factor, ASOT, cryoglobulins
Urine analysis and microscopic examination	serum electrophoresis, immunoglobulins, serum free light chains
Renal ultrasound	hepatitis and HIV serology
Chest X-ray	
**Follow-up after Acute Kidney Injury**	
Nephrology referral within 3 months after AKI episode	SCr, urea, and proteinuria
	Medication reconciliation
	Education on nephrotoxic avoidance
	Strategies to prevent CKD progression

ANA: antinuclear antibodies; ANCA: antineutrophil cytoplasmic antibodies; anti-GBM: anti-glomerular basement membrane; anti-dsDNA: anti-double stranded DNA; ARB: angiotensin receptor blocker; ASOT: antistreptolysin O titer; CKD: chronic kidney disease; RRT: renal replacement therapy.
